# Measuring patient's expectation and the perception of quality in LASIK services

**DOI:** 10.1186/1477-7525-7-63

**Published:** 2009-07-10

**Authors:** Deng-Juin Lin, Ing-Cheau Sheu, Jar-Yuan Pai, Alex Bair, Che-Yu Hung, Yuan-Hung Yeh, Ming-Jen Chou

**Affiliations:** 1 Institute of Medicine, Chung Shan Medical University, Taichung, Taiwan; 2 Center for General Knowledge Education, Chung Shan Medical University, Taiwan; 3 Department of Health Policy and Management, Chung Shan Medical University, Taichung, Taiwan; 4 Center for Education and Research on Geriatrics and Gerontology, Chung Shan Medical University, Taichung, Taiwan; 5 Chung Shan Medical University Hospital, Taichung, Taiwan; 6 Bair's eye center, Taichung, Taiwan; 7 School of Statistics, Capital University of Economics and Business,Taiwan; 8 StatSoft Holdings, Inc., Taiwan Branch

## Abstract

**Background:**

LASIK is the use of excimer lasers to treat therapeutic and refractive visual disorders, ranging from superficial scars to nearsightedness (myopia), and from astigmatism to farsightedness (hyperopia). The purposes of this study are to checking the applicability and psychometric properties of the SERVQUAL on Lasik surgery population. Second, use SEM methods to investigate the loyalty, perceptions and expectations relationship on LASIK surgery.

**Methods:**

The method with which this study was conducted was questionnaire development. A total of 463 consecutive patients, attending LASIK surgery affiliated with Chung Shan Medical University Eye Center, enrolled in this study. All participants were asked to complete revised SERVQUAL questionnaires. Student t test, correlation test, and ANOVA and factor analyses were used to identify the characters and factors of service quality. Paired t test were used to test the gap between expectation and perception scores and structural equation modeling was used to examine relationships among satisfaction components.

**Results:**

The effective response rate was 97.3%. Validity was verified by several methods and internal reliability Cronbach's alpha was > 0.958. The results from patient's scores were very high with an overall score of 6.41(0.66), expectations at 6.68(0.47), and perceptions at 6.51(0.57). The gap between expectations and perceptions was significant, however, (t = 6.08). Furthermore, there were significant differences in the expectation scores among the different jobs. Also, the results showed that the higher the education of the patient, the lower their perception score (r = -0.10). The factor loading results of factor analysis showed 5 factors of the 22 items of the SERVQUAL model. The 5 factors of perception explained 72.94% of the total variance there; and on expectations it explained 77.12% of the total variance of satisfaction scores.

The goodness-of-fit summary, of structure equation modeling, showed trends in concept on expectations, perceptions, and loyalty.

**Conclusion:**

The results of this research appear to show that the SERVQUAL instrument is a useful measurement tool in assessing and monitoring service quality in LASIK service, and enabling staff to identify where improvements are needed, from the patients' perspective. There were service quality gaps in the reliability, assurance, and empathy. This study suggested that physicians should increase their discussions with patients; which has, of course, already been proven to be an effective way to increase patient's satisfaction with medical care, regardless of the procedure received.

## Background

LASIK is the use of excimer lasers to treat therapeutic and refractive visual disorders ranging from superficial scars to nearsightedness (myopia), astigmatism, and farsightedness (hyperopia). In the USA, more than 1.1 million LASIK procedures were performed in 2003 out of a total of 3.0 million worldwide. There is much research identifying LASIK as a state of the art procedure currently being used to correct all levels of myopia, astigmatism, and hyperopia [1,2], and these problems are corrected with less haze and earlier stabilization of visual acuity than other methods of treatment, or if left untreated [3].

### Service quality

Gronroos (1984) argued that there are two distinct constituents of service quality, the technical and the functional. In the health care field, technical quality focuses on the technical accuracy of the medical diagnosis and procedures, while functional quality is the manner in which the health care was provided. However, in the context of health care, the technical quality was difficult to evaluate for consumers [4], and this resulted in most patients evaluating health care based on the functional aspects alone. Parasuraman [5] defined service quality as the difference between customer expectations and customer perceptions. When expectations are greater than perceptions a service quality gap exists.

Patient satisfaction should be interpreted carefully, due to the lack of theoretical foundations on which the concept of satisfaction and measurement are based [6]. Patients are an active consumer of health care services rather than merely passive recipients [7]. The validity and reliability of many studies on health care consumer satisfaction have been questioned [8].

The original PZB model [5] identified 10 determinants of service quality. The subsequently developed SERQUAL [9] recast the 10 determinants into five specific components: tangibles, reliability, responsiveness, assurance, and empathy. These five components are a factor analysis of the 22-items scale. Measuring quality of care from the patient's perspective has been increasingly used and accepted in health care research [10-12]. One study used the SERVQUAL service quality to measure the expectation and perception of Greek patients on dental health care [5,9,13,14]. Another study used a refined version of SERVQUAL to measure patient satisfaction in health services in Bangladesh [15] and the results found that the "tangible" factor was the most important factor in health service quality. Patient satisfaction, however, has rarely been considered in cataract surgery [16-18] and few studies have addressed the role of the hypothesized determinants of patient satisfaction.

### Purpose

The purposes of this study are to checking the applicability and psychometric properties of the SERVQUAL on Lasik surgery population. Second, SEM methods are used to investigate the loyalty, perceptions and expectations relationship on LASIK surgery.

## Methods

### Patients and Institution

466 out of 476 consecutive patients undergoing day-stay LASIK surgery at Chung Shan Medical University Bair's Eye Center in Taichung, Taiwan were invited to participate in this study, when patients finished the follow up visit after their operation from June 2006 to May 2007. Patients who declined the questionnaire indicated it was due to personal time limitations. The Sample Eye Center is one of the largest eye centers in central Taiwan and serves a large number of eye disease patients, drawn from the two million person population of metropolitan Taichung. The eye center has four full time ophthalmologists and offers LASIK procedures, including Wavefront LASIK. Like most technology-driven fields, LASIK continues to evolve as system vendors and surgeons look for new ways to apply technology to improve surgical outcomes. The most promising of these new approaches is called Optimized Aspherical Transition Zone, topo-guided wavefront-guided LASIK combined with the Torsion Error Detection (TED), or simply, Wavefront LASIK (also known as Custom Ablation). The procedure of LASIK contains three major steps, they are: preoperative evaluation, operation, and a follow up after the operation. In the first component, preoperative evaluation: patients have to under go seven preoperative diagnostic tests: auto refraction, visual acuity, pneumotonometry, slit lamp exam, Topography, Pachometery. In the second component, operation, major operations are conducted. And in the third component, follow up after operation, patients have to under go 5 post operation diagnostic tests: auto refraction, visual acuity, slit lamp exam, retina examination, and topography.

### Research Design

This study used the adapted and revised SERVQUAL conceptual model of service quality in conjunction with the SERVQUAL questionnaire to measure the expectation and perception of LASIK patients.

The SERVQUAL instrument was designed to measure service quality using both the gap concept and service quality dimensions. The original SERVQUAL contains 22 pairs of the Likert scale on five service quality dimensions and are defined as follows:

1. Tangibles: The appearance of physical facilities, equipment, appearance of personnel, and communication materials.

2. Reliability: The ability to perform the promised service dependably and accurately.

3. Responsiveness: The willingness to help customers and provide prompt service.

4. Assurance: The knowledge and courtesy of employees and their ability to inspire trust and confidence.

5. Empathy: The caring, individualized attention the firm provides to its customers

The questionnaire (see Appendix) was composed of four parts and used 7 points on the Likert scale (strongly disagree = 1 to strongly agree = 7). The first part, the perception and expectation component, (quality gap) is composed of 22 paired items on service quality. The second part is three items on loyalty which are overall satisfaction, willingness to revisit, and a willingness to recommend to friend[19,20]. These items which measure what we termed customer loyalty could serve as anchor items to examine the criterion-related validity of the scale. The third part of these questionnaires is composed of the patient's background data, such as sex, age, job, level of education achieved, date of LASIK surgery, and the outcome of the LASIK surgery. The fourth part is an open area where patients can write their comments and/or any ideas about the service they received.

### Reliability and Validity

#### Internal consistency and reliability

The expectation and perception scale had an alpha coefficient of > 0.958 (Table 1). Also, the correlations in "item to total" were all from 0.36 to 0.90.

**Table 1:** Reliability and Paired t test

	Mean	SD		Cronbach's alpha
Expectation	6.68	0.47	t = 6.08*	0.958
Perception	6.51	0.57		0.967

Note 1: * means significant at 0.05 level

#### Content validity

Content validity of the questionnaire was further confirmed by 3 ophthalmologists and 2 management specialists. The validity was also verified through several literature reviews on the SERVQUAL service model [5,9,13-15,19].

#### Construct validity

On the basis of a review of the literature, the latent construct of patient expectations and perceptions of quality was theorized to be multidimensional. The factor analysis (Table 2) identified five dimensions of expected and perceived quality [21,22].

**Table 2:** Factor loading of patients' satisfaction

	Loadings						Loadings				
			
Expected	Factor 1	Factor 2	Factor3	Factor 4	Factor 5	Perceived	Factor 1	Factor 2	Factor 3	Factor 4	Factor 5
E1			**0.79**			P1	**0.80**				
E2			**0.80**			P2	**0.78**				
E3			**0.81**			P3	**0.68**				
E4			**0.77**			P4	**0.64**				
E5		**0.86**				P5					**0.68**
E6		**0.86**				P6					**0.75**
E7		**0.87**				P7					**0.77**
E8		**0.85**				P8					**0.67**
E9		**0.86**				P9					**0.65**
E10	**0.86**					P10				**0.68**	
E11	**0.88**					P11				**0.81**	
E12	**0.84**					P12				**0.78**	
E13	**0.80**					P13				**0.70**	
E14				**0.85**		P14			**0.83**		
E15				**0.85**		P15			**0.81**		
E16				**0.81**		P16			**0.87**		
E17				**0.86**		P17			**0.82**		
E18					**0.72**	P18		**0.70**			
E19					**0.66**	P19		**0.78**			
E20					**0.82**	P20		**0.85**			
E21					**0.75**	P21		**0.79**			
E22					**0.79**	P22		**0.77**			

Eigenvalue	8.85	2.69	2.04	1.88	1.51	Eigenvalue	9.89	2.01	1.84	1.25	1.06

% of Variance	40.21%	12.23%	9.27%	8.55%	6.86%	% of Variance	44.95%	9.13%	8.36%	5.69%	4.80%

#### Criterion-related validity and predictive validity

**Figure 1:**
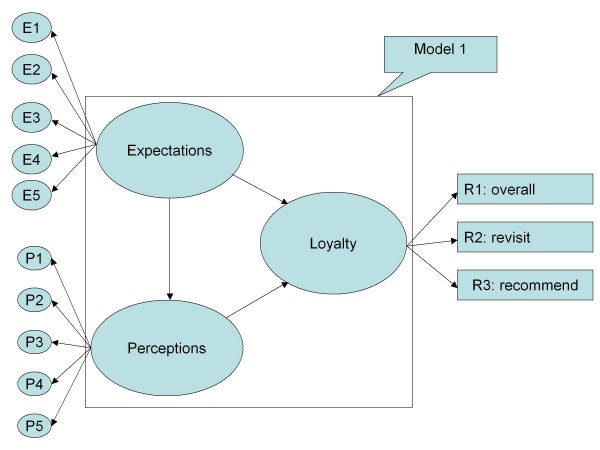
**SEM on patients' satisfaction model 1**. Indicated the initial SEM patients' satisfaction model.

Criterion-related validity and predictive validity, identified in Table 3 and Figure 1, indicated that the expected and perceived quality scale was associated with loyalty which included overall satisfaction, willingness to revisit, and willingness to recommend to friends [20]. Also, the goodness of fit indices provides model validity [23].

**Table 3:** Goodness-of-fit summary for patients' satisfaction model

	χ^2^	df	χ^2^/df	RMSEA	PGI	APGI	GFI	AGFI	Bollen's Rho	BC Index
Model 1	268.516	62	4.33	0.085	0.914	0.873	0.897	0.849	0.921	0.866
Model 2	269.358	63	4.28	0.084	0.914	0.876	0.91	0.87	0.922	0.863

Note: BC Index: Browne-Cudeck Cross Validation Index

#### Convergent validity

Bollen's Rho coefficient equal to 0.921 and 0.922 which are greater than 0.70.

### Statistic analysis

The software STATISTICA^® ^version 7.1 was used for the statistics analysis spread through out this research, including the Structural equation modeling (SEM), Student t test, correlation test, and ANOVA used to test the overall satisfaction with patient's characteristics. Factor analyses [24], which are a data-reduction technique, were used to determine the number and nature of factors of service quality that underlie a set of variables. The principal axis method was used to extract all factors that had eigenvalues greater than 1 and, therefore, could explain a significant amount of the total variance. Scree tests were used to identify the number of factors to retain. Paired t tests were used to test the gap between expectation and perception scores. Structural equation modeling was used to examine relationships between satisfaction components. The (alternative) hypotheses were stated as follows:

H1: Perceptions are positively correlated with expectations.

H2: Loyalty is positively correlated with perceptions.

H3: Loyalty is positively correlated with expectations.

The hypotheses were tested via SEM using the STATISTICA^®^7.1 package. The parameters estimated were the regression coefficients in this structural equation part of the SEM. The assessment of model adequacy was based on the following goodness-of-fit criteria: Normed chi-square (χ^2^/df) < 3, root mean square error of approximation (RMSEA) < 0.08, population gamma index (PGI), adjusted population gamma index (APGI), goodness-of-fit (GFI), adjusted goodness-of-fit (AGFI), and Bollen's Rho > 0.8 [25]. Browne-Cudeck Cross Validation Index close to 0.9 is considered a good fit.

## Results

466 out of 476 patients agreed to fill out the questionnaire after they finished the post-operation assessment. The director of staff facilitated the questionnaire request. Among the returned questionnaires, three of them were not complete; therefore, 463 (97.3%) copies were considered effective responses. The patient's characteristics are presented in Table 4. The mean (SD) age was 29.0 (5.5) years, 327 (70.62%) patients were female, 383 (87.8%) patients had a college degree or higher.

**Table 4:** Patients' Characteristics

Characteristics		Range
No. of patients	463	
Age, mean (SD)	29.03 (5.52)	17–59
No. of women (%)	327 (70.62%)	
Education degree		
	Junior high	2
	Senior high	78
	College	336
	Graduate	47

The results of the scores of patients showed very high on the overall satisfaction 6.41 (0.66), expectations 6.68 (0.47), and perceptions 6.51 (0.57) in Table 5 and Table 1.

**Table 5:** Tests on the overall satisfaction with patients' characteristics

No. of patients	463			
Overall Satisfaction		6.41(0.66)	Range 3–7	
Sex	Male	6.40(0.70)	df = 461	
	Female	6.44(0.57)	t = 0.65 (NS)	
Age			r = 0.07 (NS)	
Job (ANOVA)	SS between = 2.459	MS = 0.351	F = 0.80 (NS)	df = 7
	SS within = 199.57	MS = 0.439		df = 455
Education degree			r = -0.03(NS)	

In Table 5, the student t test on sex showed there was no difference on the overall satisfaction between male and female, also the correlation test revealed there was no significant relationship between age, job, or education, and with the patient's overall satisfaction.

In Table 6, the student t test on gender showed females have higher expectation levels than their male counterparts. However, there was no significant difference in perception and loyalty scores. Furthermore, the correlation test revealed there was no significant relationship on age items. ANOVA results showed there were significant differences in expectation scores between various occupations. A further LSD test on Table 7 showed yet more details. In expectations, public service, students and others have lower scores, where as house keepers and service industry workers have higher scores. The most interesting correlation was in the level of education achieved; the higher the degree of education, the lower the scores in perception (r = -0.10).

**Table 6:** Tests on the satisfaction with patients' demographics

Gender	e1–e22	Female	6.73 (0.37)	t = 3.65*
		Male	6.56 (0.64)	
	p1–p22	Female	6.50 (0.60)	t = 0.48 (NS)
		Male	6.53 (0.51)	
	Loyalty	Female	6.48 (0.64)	t = 0.33 (NS)
		Male	6.50 (0.52)	
Age	e1–e22			r = 0.08 (NS)
	p1–p22			r = 0.07 (NS)
	Loyalty			r = 0.09 (NS)
Job	e1–e22			F = 2.69*
	p1–p22			F = 1.30 (NS)
	Loyalty			F = 1.12 (NS)
Education degree	e1–e22			r = -0.02 (NS)
	p1–p22			r = -0.10*
	Loyalty			r = -0.05 (NS)

Note 1: * means significant at 0.05 level

Note 2: NS means not significant at 0.05 level

**Table 7:** LSD test on the JOB and Expectation

	{1} M = 6.81	{2} M = 6.60	{3} M = 6.68	{4} M = 6.68	{5} M = 6.74	{6} M = 6.51	{7} M = 6.82	{8} M = 6.62
{1}		0.060	0.186	0.215	0.770	**0.010**	0.965	0.065
{2}			0.331	0.398	0.568	0.381	**0.009**	0.828
{3}				0.981	0.801	0.051	**0.035**	0.390
{4}					0.798	0.083	0.072	0.474
{5}						0.357	0.745	0.616
{6}							**0.000**	0.241
{7}								**0.006**

The loading results of factor analysis in Table 2 showed 5 factors in the SERVQUAL model perceived (explained 72.94% of total variance) and expected (explained 77.12% of total variance) satisfaction scores. Although some of the items showed a little overlap, the 22 items were relatively well distributed over the five factors. In addition, the eigenvalues criteria and Scree tests further confirmed these 5 factors.

In order to see whether there were gaps between the patient's expectations and perceptions, paired t tests were conducted in Table 1. The results demonstrated that patients had a higher score in expectations than in perceptions, which, of course, means there was a quality gap between them.

**Figure 2:**
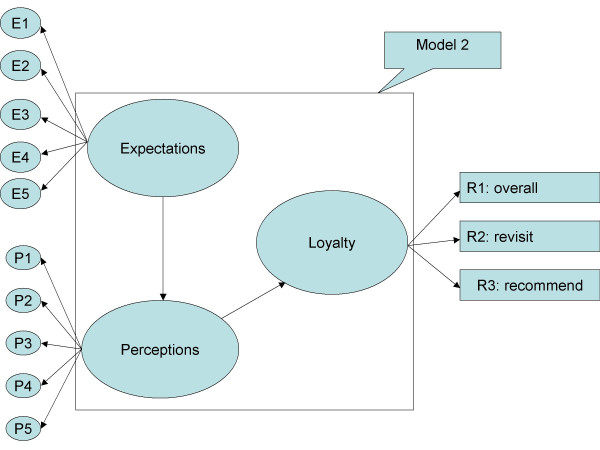
**SEM on patients' satisfaction model 2**. Indicated the final model which shows the perceptions are positively correlated with expectations. Also, loyalty is positively correlated with perceptions, and, however, loyalty is positively correlated with expectations was rejected.

Structural Equation Modeling (SEM) of the patient's satisfaction was undertaken in the goodness-of-fit measuring model. The SEM approach was considered appropriate for estimating among multiple dependent and independent latent variables and providing a better model for the complex relationships among satisfaction components [26]. The goodness-of-fit summary of structure equation modeling on Figure 1, Figure 2, Table 3 and Table 8 showed the direction and concept in expectations, perceptions, and loyalty. Table 8 showed path coefficient for Model 1 and Model 2. Since Model 1 and Figure 1 do not show adequate results, it has been revised into Model 2 and Figure 2. The revised model's results in Model 1 show adequate test results in RMSEA, PGI, APGI, GFI, AGFI, Bollen's Rho, and Browne-Cudeck Cross Validation Index. Based on the SEM results the path coefficient showed that the first two hypotheses were correct. Research Hypothesis H1 (perceptions are positively correlated with expectations) and H2 (loyalty is positively correlated with perceptions) are accepted, and however, H3 (loyalty is positively correlated with expectations) was rejected.

**Table 8:** Path Coefficients of SEM Models

Model 1			Estimate	S.E.	C.R.		Model 2			Estimate	S.E.	C.R.	
E	to	Loyalty	0.024	0.37	0.63	NS							
E	to	Perception	0.392	0.062	6.37	*	E	to	Perception	0.52	0.067	7.78	*
P	to	Loyalty	0.621	0.39	16.02	*	P	to	Loyalty	0.825	0.152	5.44	*
E	to	E5	1				E	to	E5	1			
E	to	E4	0.858	0.034	24.91	*	E	to	E4	0.86	0.036	23.82	*
E	to	E3	0.97	0.036	27.13	*	E	to	E3	0.952	0.038	25.06	*
E	to	E2	0.795	0.033	23.97	*	E	to	E2	0.791	0.035	22.71	*
E	to	E1	0.701	0.051	13.88	*	E	to	E1	0.745	0.051	14.57	*
P	to	P5	1				P	to	P5	1			
P	to	P4	0.838	0.028	30.34	*	P	to	P4	0.886	0.149	5.94	*
P	to	P3	0.89	0.029	30.21	*	P	to	P3	0.949	0.159	5.96	*
P	to	P2	0.826	0.029	28.02	*	P	to	P2	0.919	0.153	6.00	*
P	to	P1	0.612	0.031	19.55	*	P	to	P1	0.737	0.131	5.64	*
L	to	R1	1				L	to	R1	1			
L	to	R2	1.103	0.055	20.06	*	L	to	R2	0.957	0.192	5.00	*
L	to	R3	1.165	0.056	20.78	*	L	to	R3	1.053	0.202	5.22	*

Note 1: * means significant at 0.05 level.

Note 2: NS means not significant at 0.05 level.

In the comments section 31 of 463 patients wrote comments. Most of the comments were positive, such as "the service was good," and "doctors and nurses responded to questions quickly and completely." However, five patients did complain about spending too long waiting. Also some wrote that the nurses were too young and too beautiful, and that the hospital should hire individuals of middle age who would give patients an impression of stability and reliability. Another patient complained that there weren't free gifts of sun glasses and facial cosmetics. One complained that the new air freshener in the LASIK waiting area was irritating.

## Conclusion

According to results of this research, we believe that our adapted version of SERVQUAL is appropriate for evaluating the service quality of LASIK service, and shared the same conclusion's of Lin's [12], which stated SERVQUAL can be used in outpatient care and that assurance and empathy were at the top of the patient's priorities. However, the largest quality gap in this study was empathy, and differed from responsiveness in Lin's study [12]. Further validation studies in various surgeries and countries are suggested to make future cross-cultural comparisons possible.

The result of this research also confirms the loyalty, perceptions and expectations relationship on LASIK surgery by SEM methods.

One of the advantages of this research study was the high percentage of effective responses (463/476 = 97.3%), compared with 79% of similar research in Tso [20] and 48.8% in Oltedal [27], 25.6% in Bankauskaite [6], 63% response rate in Hendriks [28] which reduced the non-responded bias.

Free gifts such as sun glasses and facial cosmetics could be used in the future, in response to a patient's response and could bolster patients impressions quite cost effectively.

The results of the psychometric properties of this research on Lasik surgery population revealed that there was no difference in the patient's satisfaction scores between males and females. Also there was no significant relationship between satisfaction scores and age, job, and education. The gender aspects of these results were the same as Hall [29]. However, these results differed from former research studies, such as Sorlie's [30], Baker [31], Lledó [32] who found that female patients facing cataract surgery displayed higher expectations than their male counterparts. This could be because LASIK was a high cost (US$2,000 dollars) and totally self-paid surgery. Second, patients expected high quality services; therefore, the high scores in expectations and perceptions could compensate for the gap between genders.

The results of this research showed historically high scores in patient's expectations (6.51/7 = 93.0%), and perceptions (6.29/7 = 89.9%). Compared with research conducted in India for outpatient (n = 1837) and inpatient services (n = 611) in primary health centers and district hospitals, their scores were lower than this study and ranged from 3.63/5 = 72.6% to 3.74/5 = 74.8% [21]. Also, Lin's study [12] in solo practice and group practice, which had scores that ranged from 3.73/5 = 74.6% to 4.11/5 = 82.2%, also lower than our study. In another study, conducted in the USA, patient satisfaction scores in relation to physicians was 78.22%, also significantly lower than this study [33]. The results of this research demonstrated that the SERVQUAL instrument is a useful measurement tool in assessing and monitoring service quality in LASIK service, and enabling staff to identify where improvements are needed from the patient's perspective. There were service quality gaps in the reliability, assurance, and empathy sections. This study suggested that physicians should increase their discussions with patients. This has already been proven to be an effective way to increase patient's satisfaction with medical care regardless of the procedure received [34].

## Limitations

This research has some limitations. First, the results of the structure equation modeling on confirmatory factor analysis show that the model is not perfect since the χ^2^/df = 4.28–4.33 is higher than the criteria's 3.0 [35], in addition, more female than male patients were enrolled in this study. However, this is due to the natural population distribution of LASIK patients, i.e. women are more unwilling to wear glasses and therefore, they will have more LASIK surgery than men.

## Competing interests

The authors declare that they have no competing interests.

## Authors' contributions

DJL was responsible for primary data cleaning and analysis, ICS served as a methodologic consultant, assisted with data analysis and interpretation, and participated in manuscript editing. JYP was responsible for primary study design, manuscript drafting, statistic and interpretation, and manuscript submission. AB served as a LASIK consultant, assisted with data collection and assisted with methodology design. CYH was responsible for statistic consultant. YHY served as medical consultant. MJC served as medical consultant. All authors have read and approved this manuscript.
